# Bioinformatic Identification of Potential RNA Alterations on the Atrial Fibrillation Remodeling from Human Pulmonary Veins

**DOI:** 10.3390/ijms241310501

**Published:** 2023-06-22

**Authors:** Wataru Igarashi, Daichi Takagi, Daigo Okada, Daiki Kobayashi, Miho Oka, Toshiro Io, Kuniaki Ishii, Kyoichi Ono, Hiroshi Yamamoto, Yosuke Okamoto

**Affiliations:** 1 Department of Cardiovascular Surgery, Akita University Graduate School of Medicine, 1-1-1 Hondo, Akita 010-8543, Japan; 2 Center for Genomic Medicine, Graduate School of Medicine, Kyoto University, Shogoinkawahara-cho, Kyoto 606-8507, Japan; 3 Department of Cell Physiology, Akita University Graduate School of Medicine, 1-1-1 Hondo, Akita 010-8543, Japan; 4 Research Department, Ono Pharmaceutical Co., Ltd., Kyutaromachi, Osaka 541-0056, Japan; 5 Department of Pharmacology, Faculty of Medicine, Yamagata University, Iida-Nishi, Yamagata 990-9585, Japan

**Keywords:** atrial fibrillation, pulmonary vein, co-expression network analysis, long non-coding RNA, ion channels, inflammatory cytokines

## Abstract

Atrial fibrillation (AF) is the most frequent persistent arrhythmia. Many genes have been reported as a genetic background for AF. However, most transcriptome analyses of AF are limited to the atrial samples and have not been evaluated by multiple cardiac regions. In this study, we analyzed the expression levels of protein-coding and long noncoding RNAs (lncRNAs) in six cardiac regions by RNA-seq. Samples were donated from six subjects with or without persistent AF for left atria, left atrial appendages, right atria, sinoatrial nodes, left ventricles, right ventricles, and pulmonary veins (PVs), and additional four right atrial appendages samples were collected from patients undergoing mitral valve replacement. In total, 23 AF samples were compared to 23 non-AF samples. Surprisingly, the most influenced heart region in gene expression by AF was the PV, not the atria. The ion channel-related gene set was significantly enriched upon analysis of these significant genes. In addition, some significant genes are cancer-related lncRNAs in PV in AF. A co-expression network analysis could detect the functional gene clusters. In particular, the cancer-related lncRNA, such as *SAMMSON* and *FOXCUT*, belong to the gene network with the cancer-related transcription factor *FOXC1*. Thus, they may also play an aggravating role in the pathogenesis of AF, similar to carcinogenesis. In the least, this study suggests that (1) RNA alteration is most intense in PVs and (2) post-transcriptional gene regulation by lncRNA may contribute to the progression of AF. Through the screening analysis across the six cardiac regions, the possibility that the PV region can play a role other than paroxysmal triggering in the pathogenesis of AF was demonstrated for the first time. Future research with an increase in the number of PV samples will lead to a novel understanding of the pathophysiology of AF.

## 1. Introduction

Atrial fibrillation (AF) is the most common cardiac arrhythmia. Researchers worldwide have devoted much effort to finding its etiology. Although we currently know that AF has a complex pathology, investigation of the specific molecular mechanisms of atrial fibrillation began with identifying the causative gene for familial atrial fibrillation in the last century. From the ancestry of familial AF, linkage analysis identified causal loci on the genetic map [1]. In 2003, it was reported that one of the causal genes in familial AF was due to mutations in the K^+^ channel KCNQ1 [2]. KCNQ1 abnormality was known to cause long QT syndrome [3], so this finding attracted much interest. Subsequently, many mutations in ion channel genes causing familial AF have been reported, and the genetic mutations leading to familial AF are well summarized in the review by Ragab et al. [4]. However, familial AF is a rare genetic disorder that hardly explains the pathogenesis of common AF.

For this reason, genome-wide association analysis (GWAS), a comprehensive analysis of single-gene polymorphisms (SNPs), has been repeatedly performed to explore the genetic background of the disease as a more common condition, i.e., non-familial AF. First 2007, an SNP at locus 4q25 was reported [5]. This locus locates between the *PITX2* gene and the translational region of the *C4orf32* gene, whose function is poorly understood, and its association with AF is overwhelmingly significant. Furthermore, the transcription factor *PITX2* has attracted attention in the understanding of the pathophysiology of AF [6], as it is involved in the development of the pulmonary veins (PVs) [7], the regional origin of AF. Subsequently, similar analyses have reported the association of the transcription factors *ZFHX3* [8,9] and the K^+^ channel *KCNN3* [10], and now nearly 120 loci have been reported to be associated with AF [11]. The problem is that SNPs at each locus have a low relative risk (RR) for developing AF. Most loci have an RR between 1.0 and 1.1 [12]. In other words, AF-associated loci explain the inherited susceptibility to AF, but a single SNP alone does not lead to the development of the disease.

Therefore, the inherited combination of several SNPs or the involvement of environmental factors leads to the onset of the disease. Transcriptome analysis helps elucidate the direct pathophysiology. Hitherto, oxidative stresses [13], metabolic changes [14], and inflammations [11] have been suggested from transcriptome analysis. Recent advances in sequencing technology allow the transcriptome and non-coding RNAs to be analyzed together, allowing even post-transcriptional considerations [15,16]. However, transcriptome analysis generally has been limited to human patient samples from the atria and has yet to elucidate the pathogenesis by multi-cardiac regions. In the present study, we compared six different cardiac regions of donated samples. Surprisingly, the PV was the cardiac region most affected by atrial fibrillation in gene expressions. Moreover, our results have the potential to explain that AF is induced by an accumulation of acquired environmental stresses, similar to carcinogenesis.

## 2. Results

### 2.1. General Overview of Analysis Results

**Figure 1:**
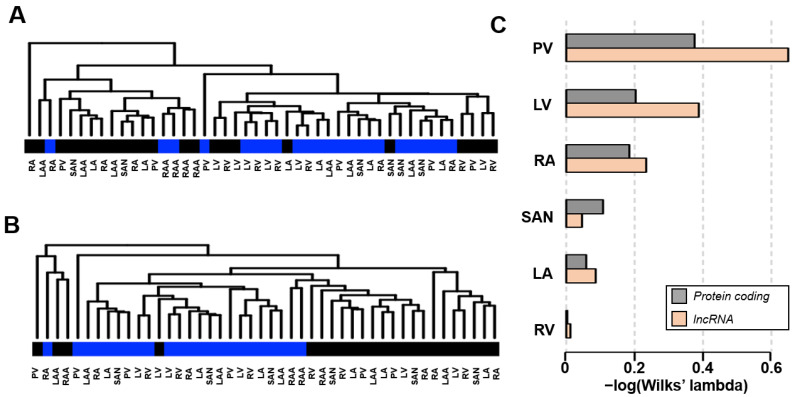
Overview of the obtained data. Twenty-three samples, each with or without AF, were compared, and (**A**) 201 coding RNA and (**B**) 212 long non-coding RNA (lncRNA) were detected as differential expression genes (DEGs). Samples were hierarchically clustered, and the origin of the samples was color coded below the dendrogram. Black codes control and blue codes AF. (**C**) Degree of separation between control and AF samples in each cardiac region. The acquired data were analyzed using principal component analysis to construct a two-dimensional space with first and second principal components. Wilks’ lambda calculates how much each cardiac region data is separated between control and AF. The smaller the value, the greater the degree of separation. The negative logarithm is scaled to present the degree of separation.

In this study, a total of 46 samples were obtained for RNA-seq; three samples each of left atrium (LA), left atrial appendage (LAA), left ventricle (LV), pulmonary vein (PV), right atrium (RA), right ventricle (RV) and sinoatrial node (SAN) were obtained from donors with or without persistent atrial fibrillation (AF); right atrial appendages (RAAs) were excised during mitral valve replacement surgery for two samples each from patients with or without AF. Patient information is summarized in Table 1. From 33535 RNAs detected by RNA-seq, statistical testing of expression differences between controls and AFs using the false discovery ratio (FDR) < 0.05 criterion resulted in 413 RNA hits, of which 201 were protein-coding, and 212 were long non-coding RNAs (lncRNAs). Using the R package, we applied the authorized method to compare two groups by computing differential expression genes (DEGs). The full results of the analysis can be viewed in Supplementary File S1. The hierarchical clustering of the DEGs in protein-coding and lncRNA are shown in Figure 1A,B, respectively. Besides the presence or absence of AF, differences in expression patterns were observed among cardiac regions. Therefore, each cardiac region was evaluated separately. Hereafter, LAA and RAA data were included in LA and RA, respectively.

First, principal component analysis was applied to the gene expression matrices of each region. Wilks’ lambda was calculated to indicate the distance between groups with or without AF in the two-dimensional space defined by the first and second principal components. Wilks’ lambda is a statistic that measures how groups are separated in space; it is expressed from 0 to 1, with the closer to 0, the greater the degree of separation. Figure 1C displays the negative logarithm of Wilks’ lambda for protein-coding (gray) and lncRNAs (orange). The most considerable difference between groups was demonstrated in PVs. Except for the SAN, differences between groups were more remarkable for lncRNAs than for protein-coding genes. It was suggested that the degree of association with the disease might differ from region to region. There is little difference in RV because the development of AF has little effect on gene expression in RV.

### 2.2. Differential Expression Genes (DEGs) in PV and LV

**Figure 2:**
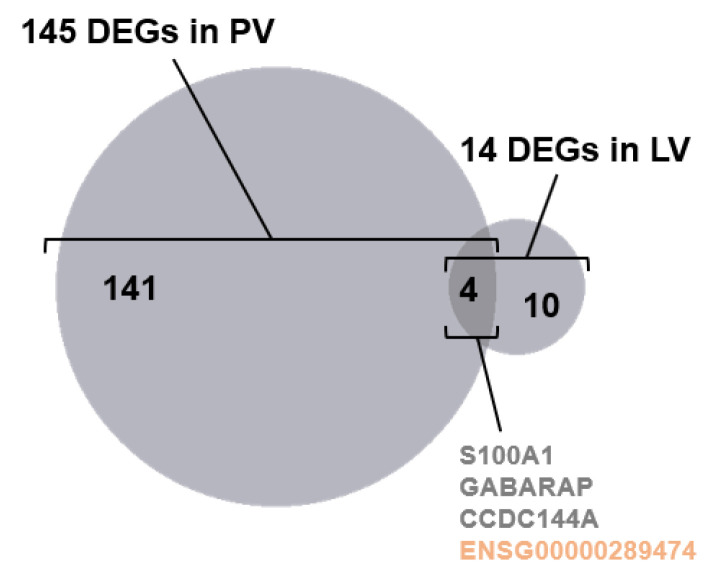
Populations of differential expression genes (DEGs) in the pulmonary vein (PV) and left ventricle (LV). 145 DEGs in PV and 14 in LV share four genes, *S100A1, GABARAP, CCDC144A,* and long non-coding *ENSG00000289474*.

**Figure 3:**
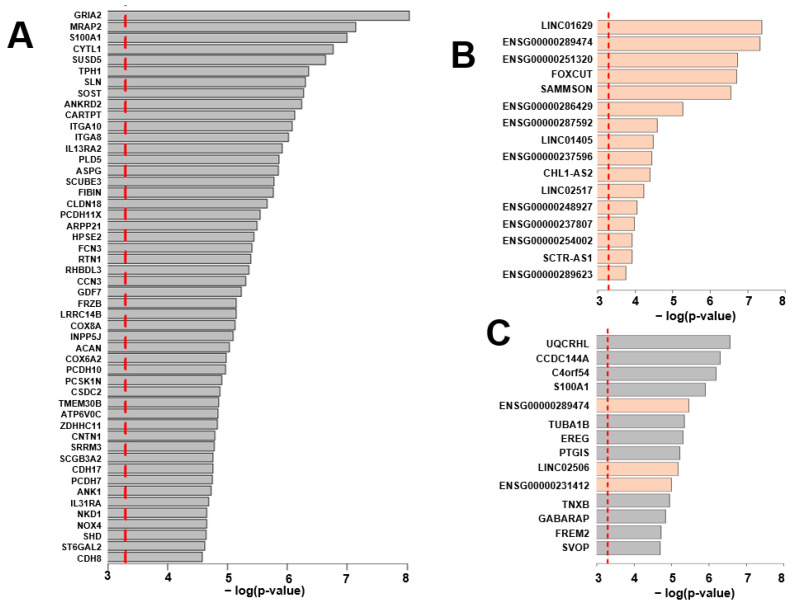
Differential expression genes (DEGs) in the pulmonary veins (PVs) and left ventricles (LVs). DEGs are presented in order of statistical significance. The red dotted line indicates a *p*-value = 0.0005. Protein-coding RNAs and long non-coding RNAs (lncRNAs) are color-coded by gray and orange, respectively. (**A**) Top 50 protein-coding DEGs in PV. (**B**) lncRNAs as DEGs in PV. (**C**) All DEGs in the left ventricle.

**Figure 4:**
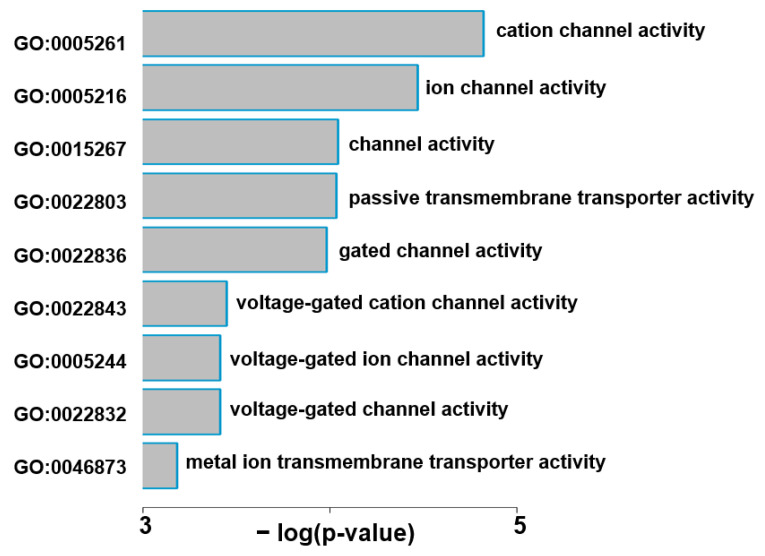
Functional annotation of differential expression genes (DEGs) in the pulmonary veins (PVs) by Gene Ontology (GO) analysis. Enriched GOs are presented in order of statistical significance. GO IDs are provided on the left. The corresponding negative logarithm of *p*-values and descriptions are in the middle and on the right, respectively. The corresponding genes are listed in Table 2.

DEG analysis was performed by cardiac region (FDR < 0.05). The results showed that 145 DEGs were identified in the PV and 14 in the LV, but not in other regions. This supports the results from the analysis of Wilks’ lambda. Four genes belonging to both PV and LV DEGs were *S100A1*, *GABARAP*, *CCDC144A*, and *ENSG00000289474* (Figure 2). Among the DEGs, the top 50 coding RNAs in PV, all lncRNAs in PV, and all DEGs in LV are listed in Figure 3A–C, according to statistical significance. The full results for DEGs in PV and LV can be viewed in Supplementary File S2. In addition, the Gene Ontology (GO) analysis was performed on the PV and LV gene sets to determine the functional annotation of protein-coding RNAs among the DEGs. Significantly enriched ontologies were identified in the PV DEGs, including “ion channel activity”. The GO IDs, descriptions, and statistics of the identified GOs are graphed in Figure 4. The gene names of the DEGs belonging to each GO are listed in Table 2.

In contrast, no significantly enriched Ontology was detected in DEGs of LV. Although most lncRNAs have poorly understood functions and cannot be generally annotated like coding RNAs, cancer-associated lncRNAs have been studied relatively well. In Table 3, lncRNAs corresponding to PV DEGs are addressed to cancer-associated lncRNAs in the database. Significant lncRNAs in PV in AF are also comparable in cancer.

### 2.3. Co-Expression Network Analysis on DEGs

**Figure 5:**
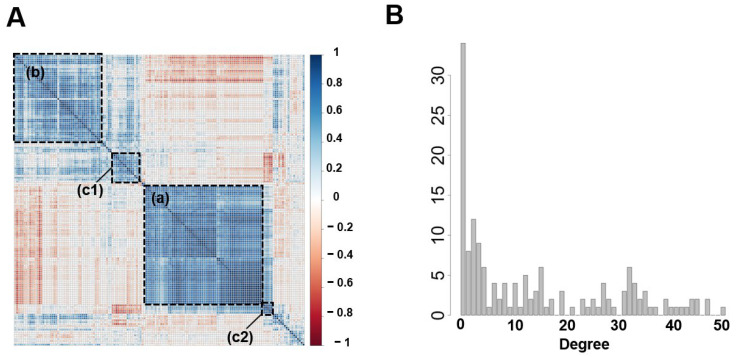
The fundamental structures of the co-expression network in differential expression genes (DEGs). (**A**) Co-expressed gene pairs are clustered and visualized as a heatmap. The color scale corresponding to the correlation coefficient is inserted on the right. Based on absolute values of a correlation coefficient of 0.8 or higher, there are three subnetworks: a compartment containing 63 genes (a) and a compartment containing 46 genes (b), as well as a compartment (c1) and (c2), which are composed of 10 and 5 genes, respectively. (c1) and (c2) are linked by negative correlation. The rest of the subnetworks are isolated genes with fewer than two nodes. Compartment (a) is represented as subnetwork A, compartment (b) as sub-network B, and compartments (c1) and (c2) as sub-network C, which are represented in Figure 6A–C, respectively. (**B**) The histogram of gene degree. The degree is proportional to the number of intergenic connections. The distribution of this histogram indicates that the network is scale-free.

**Figure 6:**
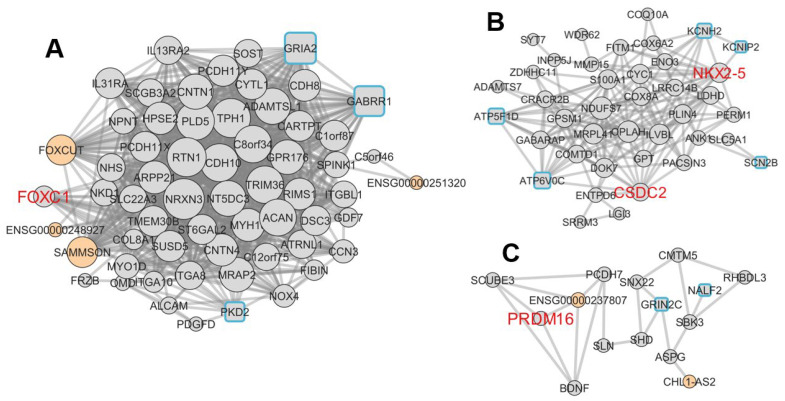
Subnetworks A, B, and C are expanded into (**A**–**C**) in this figure. The diameter of the nodes is proportional to the degree that the gene has. Coding RNAs are grey, lncRNAs are orange nodes, and square nodes indicate ion channel-related genes enriched in the GO analysis with a marine blue border. Transcription factors are highlighted in red letters. Subnetwork A consists of 62 genes and is a network of genes whose expression is up-regulated compared to controls. Subnetwork B is a network of 40 genes whose expression is down-regulated. Subnetwork C consists of five up-regulated and ten down-regulated genes. All nodes represent differential expression genes of the pulmonary vein except *PTGIS*, which is in subnetwork A.

To explore functional relationships between DEGs, co-expression network analysis was performed on 155 DEGs detected in PV and LV. This method is a valuable data analysis technique in characterizing gene function globally [17] and is also used in investigations of lncRNA function [18]. By clustering Pearson correlation coefficients between gene expression levels, compartmentalized groups of genes with correlated expressions are visualized (Figure 5A). A gene network was constructed by linking pairs of these gene groups with correlation coefficients >0.8. In Figure 5A, the largest compartment (a), followed by compartment (b), and the negatively linked compartments (c1) and (c2) are constructed as subnetworks A, B, and C. Subnetwork A, B, and C are extracted into Figure 6A–C, respectively. Figure 5B displays the distribution of gene degree (number of intergenic connections). These networks have typical gene network properties, with a few genes having many connections and most genes having few connections, referred to as scale-free [19]. Genes with a degree above 30 are listed in Table 4 as hub genes, which play essential roles in the gene network. In Figure 6, subnetwork A is a population of up-regulated 62 genes, subnetwork B is a population of down-regulated 40 genes, and subnetwork C is mixed by up-regulated five and down-regulated ten genes. Subnetworks A and C have no known genes that reportedly induce AF and have several lncRNAs, which are thought to be involved in regulating post-acquired gene expression. In contrast, subnetwork B has known AF-related genes such as *NKX2-5*, *KCNH2*, and *SCN2B* and no lncRNAs.

## 3. Discussion

Although there have been many comprehensive RNA expression analyses on atrial fibrillation (AF), most studies have limited their analysis to atrial samples on the assumption that pathological RNA alterations occur in the atria [13,14,15,16]. We are the first to perform comparisons in six different heart regions. Surprisingly, the most robust RNA alterations occurred in the pulmonary veins (PVs) among the cardiac regions (Figure 1B). Of these, coding RNAs related to ion channels are particularly enriched as RNAs influenced by AF (Figure 4). This may illustrate the basis of electrical remodeling in AF patients. In addition, the expression of the transcription factor FOXC1 was strongly up-regulated with long non-coding RNA (lncRNA) *FOXCUT* in AF patients (Figure 6A), which may be involved in structural remodeling by post-acquired gene regulation as discussed below.

### 3.1. The Priority of PV among Cardiac Regions on AF Remodeling

AF is known to be mainly triggered by PV sleeves [20]. Nevertheless, AF itself is defined as an atrial arrhythmia, and it has long been considered that substrate changes in the atria are responsible for the persistence of the arrhythmia [21]. Thus, most AF researchers believe that the initiation and maintenance of AF result from different molecular mechanisms occurring in different cardiac regions [22]. However, intense fibrosis, representing the substrate changes in AF patients, also develops in PV [23,24]. If substrate changes provide the basis for AF persistence, the atria would not be the only cardiac region contributing to AF persistence. Instead, the current analytic results indicate that biological change is more potent in PVs (Figure 1B). In our analysis, the cardiac regions with statistically significant differences between AF and sinus rhythm patients were PV and LV (Figure 2). There were 145 significant hits in the PV samples, including coding RNAs and long non-coding RNAs (lncRNAs), compared to 14 gene hits in the LV. Furthermore, samples from donors with coronary artery disease are largely unaffected in atrial and PV samples, while ventricular samples may be affected by ischemic changes in the data.

### 3.2. The Possible Basis of the Electrical Remodeling in PV in AF

Gene ontology (GO) analysis detected several ion transport-related genes from the enriched gene sets on PV, such as a subunit of mitochondrial ATPase (ATP5FD1), endoplasmic reticulum proton pump (ATP6V0C), hERG channel (KCNH2), an auxiliary subunit of BK channel (LRRC38), voltage-gated Ca^2+^ channel (CACNA1E), ion channel scaffold molecule (PKD2), β subunit of voltage-gated Na^+^ channel (SCN2B), β subunit of voltage-gated K^+^ channel (KCNIP2), a part of ion channel-type glutamate receptors (GRIA2, GRIN2c) and GABA receptors (GABRR1). 

*ATP5FD1*, *ATP6V0C*, *KCNH2*, *SCN2B*, and *KCNIP2* are essential to myocardial functions and significantly downregulated in AF samples. In addition, these cardiac function-related proteins are all on subnetwork B in Figure 6B. As noted, the co-expression network analysis was calculated from n = 46. Subnetwork B contains the transcription factor *NKX2-5*. *NKX2-5* is a well-established transcription factor that determines morphological and functional features of the myocardium [25] and is also down-regulated in AF samples. It is, thus, estimated that cardiomyocyte-like characteristics might be lost in the PVs of AF patients. Among these genes, *NKX2-5*, *KCNH2*, and *SCN2B* have already been reported as single nucleotide polymorphisms for AF [26,27,28]. Unlike mutations, polymorphisms by themselves do not induce disease and are therefore explained as a genetic background rather than a causative gene. In the present analysis, the simultaneous finding of the down-regulation of these genes is meaningful in considering the cause of the proarrhythmic state. Namely, the down-regulation of *NKX2-5* indicates a deviation from cardiac tissue, accompanied by electrical remodelings, i.e., the down-regulation of *KCNH2* and *SCN2B*, resulting in the formation of atrial fibrillation. Also, the present analysis, which does not assume genetic mutations, shares results with the analysis of congenital causes, making it reasonable to assume that the polymorphisms identified in the previous reports are responsible for the proarrhythmic state.

Other ion transport proteins belonging to subnetworks A and C are tentatively ion channel-related molecules expressed in the smooth muscle layer and the pulmonary veins’ nerve plexus.

### 3.3. Possible Classifying Post-Acquired Disturbances in AF by the Network Analysis

In the co-expression network analysis in Figure 6, subnetwork A is the extensive network with the highest number of correlated RNAs. It exhibits up-regulated expressions of the cancer-associated lncRNAs *FOXCUT*, *SAMMSON*, *ENSG00000251320*, and *ENSG00000248927*, as well as ion channels in nerve fibers, such as *GRIA2* and *GABARR1*. In addition, the expression of *FOXC1*, a *FOXCUT*-related transcript, is also up-regulated. Furthermore, prothrombotic factors, such as *PDGFD* and *ALCAM*, and molecules in the inflammatory response, such as receptors for IL-13 and 31, are also up-regulated. On the other hand, unlike subnetwork B, none of the known AF-causing genes are listed. 

Given the above, it is likely that subnetwork A reflects post-acquired pathological disturbances due to the disease rather than inherited conditions. LncRNAs are often influential in regulating acquired gene expression through epigenomic regulation and post-transcriptional and post-translational modifications [29], e.g., *FOXCUT* binds to FOXC1 mRNA and promotes protein translation [30]. The transcription factor FOXC1 is known to contribute to the formation of the heart tube outflow tract early in development in the cardiovascular research field [31]. The PV is the cardiac region that forms a part of the outflow tract [32]. However, considering the age of the donors (Table 1), the increased expression in AF may represent a pathological rather than a developmental change. As for pathological changes, FOXC1 is up-regulated in many carcinomas. It plays a unique role in promoting the epithelial–mesenchymal transition (EMT) [33]; If EMT occurs in the pulmonary veins, it is assumed that endothelial cells will be converted to fibroblasts. Some reports have already found that EMT occurs in the atria of AF patients [34,35]. Then, fibroblasts secrete cytokines and adhesion molecules that may influence the substrate changes in the patients. Indeed, *PDGE* and *ALCAM*, prothrombotic factors in subnetwork A, can be secreted by fibroblasts. Thus, fibroblasts would be expected to proliferate and the substrate alteration to progress irreversibly. Our analysis indicates that the focus of the substrate changes is the PV rather than the atria, which may be related to thrombi being more likely to occur in the left atrium (~90%) than in the right atrium [36].

*SAMMSON* is also a well-studied lncRNA initially described as up-regulated in malignant melanoma [37]. The *SAMMSON* is located 30 kilobase pairs downstream of *MITF*, a melanoma-specific transcriptional regulator, whose expression correlates with *MITF*. However, unlike the relationship between *FOXCUT* and FOXC1, *SAMMSON* does not directly contact MITF but epigenetically regulates transcription factors influencing MITF expression. While MITF is a melanoma-specific factor, *SAMMSON* has also been observed to be up-regulated in thyroid cancer [38] and glioblastoma [39]. What genes *SAMMSON* couples to in the pulmonary veins of patients with AF is a subject for future research.

Furthermore, lncRNAs can imply regulation of gene expression by environmental factors, but the specific environmental factors themselves are often unknown. On the other hand, increased cytokine receptor expression has been detected in sub-network A, indicating that one of the environmental factors is inflammation or oxidative stress. Both *IL13RA2* and *IL31RA* are receptor genes for inflammatory cytokines, IL13 and IL31. These cytokines play essential roles in acquired immunity [40,41]. In recent years, *IL13RA2* has also been reported as a marker for several cancer cells [42,43,44,45].

Although few studies have investigated the analogies between AF and cancer, both diseases are characterized by a high prevalence in the elderly and an increased incidence with age. Previous studies have revealed that the transcriptome profile of age-related changes is characterized by increased inflammation-related genes, among other features [46]. Therefore, age-related changes in molecular profiles may contribute to the risk of age-related diseases through increased inflammation. On the other hand, we do not know of any other transcriptomic studies of human cardiac PV, so we do not know the relationship between the molecules identified in this study and aging. Further profiling of pulmonary veins will lead to a better understanding of age-related diseases.

In this way, it has been explained that sub-network A represents environmental factors and sub-network B myocardial functional factors. In contrast, sub-network C is a mixture of up-and-down-regulated genes and a few genes with a low degree, so biological considerations are challenging. Increasing the number of significant genes would be necessary to make sense of these complementary networks by increasing the sample size.

### 3.4. Limitations

The primary limitation of the current study is the limited size of sample numbers. The present analysis has been performed on three AF samples and three normal heartbeat samples. It is not guaranteed that the results would be replicated if the sample size were huge or if the property of the domination were aligned by sex, age, or race. However, our study focuses on comparing multiple cardiac regions, and it is not easy to include more replicates. In the RNA-seq research community, a sample size of n = 3 is often considered a minimum requirement. This is even clearly stated in the submission guidelines of specialized omics journals such as Genomics (https://www.sciencedirect.com/journal/genomics, accessed on 3 May 2023). Thus, our study design is considered acceptable. Besides, we took advantage of the properties of omics data to make our analysis more robust with the GO and co-expression gene network analyses. GO analysis uses genome-wide information and provides more reliable biological findings even with small sample sizes. The gene network analysis identifies essential genes using genome-wide information from all n = 46 samples. 

Second, we are unable to analyze molecules regulated by micro RNAs because our analysis depends on detecting poly A tails. Some connexin genes, one of the AF-related gene family [47,48,49], are regulated by micro RNAs [50,51], but unfortunately, post-transcriptional modifications through micro RNAs are outside the focus of this study. Moreover, our hypothesis discussed above should also be confirmed in vitro to see how endothelial cells transform into fibroblasts in the future.

## 4. Materials and Methods

### 4.1. RNA Extractions from Donated Hearts and Right Atrial Appendage Collections

Human heart samples from subjects with or without persistent atrial fibrillation (AF) for left atrium (LA), left atrial appendage (LAA), right atrium (RA), sinoatrial node (SAN), left ventricle (LV), right ventricle (RV), and pulmonary vein (PV) were purchased from AnaBios Corporation (San Diego, CA, USA), which provides heart organs through the organ procurement organization of the USA in compliance with the Health Insurance Portability and Accountability Act. All personal information of donors is protected. Donated hearts were separated into SAN, LA, LAA, RA, SAN, LV, RV, and PV. These samples were frozen in RNAlater (Thermo Fisher Science, Waltham, MA, USA) at −20 °C. Our laboratory extracted RNAs using QIAGEN RNeasy mini columns (QIAGEN, Venlo, Netherland). Right atrial appendage (RAA) samples were obtained from four patients undergoing mitral valve surgery. Two of these patients had permanent atrial fibrillation; the other two were in sinus rhythm with no history of atrial fibrillation. After excision, all tissue specimens were frozen in liquid nitrogen and stored at −80 °C for later use. RNAs were extracted using ISOGEN II (NIPPON GENE CO., LTD. Kandashiki-machi, Tokyo, Japan).

### 4.2. RNA-Seq and Data Processing on the Samples

The high-throughput sequence was demonstrated with NovaSeq 6000 (Illumina, San Diego, CA, USA). First, extracted RNAs were purified by poly(A) capture. Resultant mRNAs were then fragmented and reverse-transcribed into single-stranded complementary DNAs (cDNAs). Subsequently, cDNAs were double-stranded by a DNA polymerase. Deoxy UTPs (dUTPs) were mixed in nucleotide materials during the polymerase reactions. Both double-stranded DNA (ds DNA) ends were ligated to a 13 bp adapter sequence. Both double-stranded DNA (ds DNA) ends were ligated to a 13 bp adapter sequence. Next, the ds DNAs were subjected to PCR amplification to prepare the multi-sized DNA library. NovaSeq Control software v1.4.0 analyzed the sequencing runs and tag sequences classified each read in the raw sequencing data. fastp software (version 0.12.4) was used for read quality control and adapter removal [52]. Reads were aligned using STAR software (version 2.7.0a) [53]. The fasta and gtf files of GRCh38/release105 obtained from the Ensemble database were used as reference genome and gene annotation information. The count values of each gene were quantified from the alignment results by featureCounts software (see [54]). We calculated the Transcripts Per Kilobase Million (TPM) values based on the calculated gene lengths and gene counts [55,56]. The current study targeted eight heart regions: left atrium (LA), left atrial appendage (LAA), left ventricle (LV), pulmonary vein (PV), right atrium (RA), right atrial appendage (RAA), right ventricle (RV), and sinoatrial node (SAN). Twenty-three samples from sinus rhythm (SR) and 23 samples from AF patients, a total of 46 samples of RNA-seq data, were used for analysis. Of these, the 21 control samples other than RAA are the same as in the previous report [57] and have been validated by a different experiment (GSE112339 in Gene Expression Omnibus (GEO) database). The TPM values were transformed by log2(intensity + 1) and used in subsequent analyses as gene expression intensity. Genes with zero TPM values in all 46 samples were excluded from the analysis. Protein coding genes and long non-coding RNA (lncRNA) genes were extracted based on gene feature format files provided by the Ensemble database in the same version used for alignment. Finally, 33,535 genes were included in this analysis, including 18,899 protein-coding genes and 14,636 lncRNA genes. The obtained data were subsequently analyzed in the following order: (1) differential expression gene (DEG) analysis, (2) multivariate analysis-of-covariance by manova function in R to calculate Wilks’ lambda, (3) coding RNA annotation by gene ontology (GO) analysis, (4) cancer-related lncRNA annotation, (5) co-expression network analysis.

### 4.3. Differential Expression Gene (DEG) Analysis

DEG analysis between SR and AF was performed using TCC (an acronym for Tag Count Comparison) in the R package. Here, the trimmed mean of M values (TMM) and edgeR methods were employed for normalization and testing, respectively [58]; genes with FDR < 0.05 were identified as significant DEGs. Gene Ontology (GO) analysis was performed using R’s clusterProfiler package to determine the GO-associated gene set (Benjamini-Hochberg adjusted *p*-value < 0.05) [59]. In the GO analysis, all genes included in the analysis were set to background. The R package’s org.Hs.eg.db (version 3.13.0) was used as the GO and gene annotation database. The list of transcription factors in humans was obtained from AnimalTFDB4.0 [60]. A gene co-expression network analysis was performed to perform a functional analysis of the identified DEGs. Pearson correlation coefficient matrices were calculated for DEGs. A gene network was constructed by connecting gene pairs with absolute values of correlation coefficients >0.8. The Cytoscape software (version 3.9.1, https://cytoscape.org/) visualizes the network. 

### 4.4. Clustering and Heatmap Visualization

Clustering and heatmap drawings of the samples were applied to the gene expression intensity matrix. Hierarchical clustering of samples was obtained by applying the dist and hclust functions in R with default settings. The heatmap.2 function and dendextend packages [61] in the gplots package of the R were used for heatmap and clustering drawing (https://CRAN.R-project.org/package=gplots). Each expression data was standardized before the heatmap drawing. 

## 5. Conclusions

At least, the current study suggests two findings: (1) the pulmonary veins (PVs) were the regions with the most intensive gene expression alterations by the atrial fibrillation (AF) incidence. Therefore, the PV region should be focused more on researching the etiology of both paroxysmal and chronic AF in the future. (2) the AF remodeling is attributable to a post-transcriptional genetic regulation similar to carcinogenesis, e.g., the FOXCUT-FOXC1 axis, SAMMSON, and IL13RA2. 

Thus, our analytic results may suggest that rather than searching the inborn genetic backgrounds, a healthy lifestyle, such as smoking cessation and exercise, is a more protective strategy against AF progression in analogy with cancer prevention. This is because that familial AF is a sporadic disease, and the relative risk of AF-associated SNPs is small enough. Future research with an increase in the number of PV samples will lead to a novel understanding of the pathophysiology of AF.

## Figures and Tables

**Table 1:** Patient information of donated or collected samples. SR and AF indicate sinus rhythm and persistent atrial fibrillation, respectively. For sex, M and F indicate male and female, respectively. For medical history: HD; heart disease, HT; hypertension, DM; diabetes, AF; atrial fibrillation, MR; mitral regurgitation, TR; tricuspid regurgitation, CAD; coronary artery disease, HCH; hypertensive cardiac hypertrophy. For heart samples: SAN; sinoatrial node, LA; left atrium, LAA; left atrial appendage, RA; right atrium, LV; the left ventricle, RV; the right ventricle, PV; pulmonary vein, RAA; right atrial appendage.

NO.	AF/SR	Age	Sex	Strain	Tabaco	Alcohol	HD	HT	DM	Cancer	Samples
**1**	SR	52	M	Caucasian	Yes	Yes	No	No	No	No	SAN, LA, LAA, RA, LV, RV, PV
**2**	SR	46	M	Filipino	Yes	Yes	No	medication	medication	No	SAN, LA, LAA, RA, LV, RV, PV
**3**	SR	23	F	Caucasian	Yes	No	No	No	No	No	SAN, LA, LAA, RA, LV, RV, PV
**4**	SR	50	M	Japanese	Yes	Yes	MR, TR	medication	No	No	RAA
**5**	SR	50	M	Japanese	Yes	Yes	MR	No	No	No	RAA
**6**	AF	58	M	Caucasian	Yes	Yes	CAD, AF	No	No	No	SAN, LA, LAA, RA, LV, RV, PV
**7**	AF	54	M	Caucasian	Yes	Yes	AF	No	No	No	SAN, LA, LAA, RA, LV, RV, PV
**8**	AF	58	F	Caucasian	Yes	Yes	AF	medication	No	No	SAN, LA, LAA, RA, LV, RV, PV
**9**	AF	59	M	Japanese	Yes	Yes	MR, TR, AF	medication	No	No	RAA
**10**	AF	70	F	Japanese	No	Yes	MR, HCH AF	medication	No	No	RAA

**Table 2:** Enriched gene sets detected by gene ontology (GO) analysis in the pulmonary veins (PVs). The Detected GOs are on the left, and corresponding gene symbols are on the right. Most genes are significantly downregulated in PV of AF patients except GRIA2, GABRR1, and PKD2.

Descriptions	Gene Symbols
**cation channel activity**	*GRIA2, ATP5F1D, ATP6V0C, KCNH2, NALF2, LRRC38, CACNA1E, PKD2, SCN2B, KCNIP2, GRIN2C*
**ion channel activity**	*GRIA2, GABRR1, ATP5F1D, ATP6V0C, KCNH2, NALF2, LRRC38, CACNA1E, PKD2, SCN2B, KCNIP2, GRIN2C*
**channel activity**	*GRIA2, GABRR1, ATP5F1D, ATP6V0C, KCNH2, NALF2, LRRC38, CACNA1E, PKD2, SCN2B, KCNIP2, GRIN2C*
**passive transmembrane transporter activity**	*GRIA2, GABRR1, ATP5F1D, ATP6V0C, KCNH2, NALF2, LRRC38, CACNA1E, PKD2, SCN2B, KCNIP2, GRIN2C*
**gated channel activity**	*GRIA2, GABRR1, KCNH2, NALF2, LRRC38, CACNA1E, PKD2, SCN2B, KCNIP2, GRIN2C*
**voltage-gated cation channel activity**	*KCNH2, LRRC38, CACNA1E, PKD2, KCNIP2, GRIN2C*
**voltage-gated ion channel activity**	*KCNH2, LRRC38, CACNA1E, PKD2, SCN2B, KCNIP2, GRIN2C*
**voltage-gated channel activity**	*KCNH2, LRRC38, CACNA1E, PKD2, SCN2B, KCNIP2, GRIN2C*
**metal ion transmembrane transporter activity**	*SLC22A3, KCNH2, NALF2, LRRC38, CACNA1E, PKD2, SCN2B, KCNIP2, GRIN2C, SLC5A1*

**Table 3:** Long non-coding RNAs (lncRNAs) annotation of atrial fibrillation-induced alterations in pulmonary veins based on cancer-related lncRNAs. Sixteen significant lncRNAs in PV were expressed on a log2 scale. Related carcinomas are listed based on two databases. (http://www.bio-bigdata.net/lnc2cancer/index.html, and http://fcgportal.org/TCLA/index.php, accessed on 1 February 2023). Red or blue letters and numbers indicate the increase or decrease in expression in the related disorder, respectively. GBM, glioblastoma; HCC, hepatocellular carcinoma; MME, malignant melanoma; NCP, nasopharyngeal carcinoma; GAC, gastric adenocarcinoma; BLBC, basal-like breast cancer; ESCC, esophageal squamous cell carcinoma; OSCC, oral squamous cell carcinoma; KIRC, kidney renal clear cell carcinoma; LUSC, lung squamous cell carcinoma; HNSC, head and neck squamous cell carcinoma; PRAD, prostate adenocarcinoma; BRCA, breast invasive carcinoma; COAD, colon adenocarcinoma; NA, not applicable.

Unique Names/Ensembl IDs	Fold Changes (log2) in PV in AF	Expressional Changes in Extra-Cardiovascular Carcinoma
*SAMMSON*	9.29	GBM, HCC, MME
*FOXCUT*	8.41	NCP, GAC, BLBC, ESCC, OSCC
*ENSG00000251320*	5.99	KIRC, LUSC, HNSC
*ENSG00000248927*	4.16	KIRC, PRAD
*ENSG00000237807*	2.46	BRCA, COAD, PRAD
*ENSG00000286429*	−4.73	NA
*SCTR-AS1*	−3.60	LUSC
*ENSG00000289474*	−9.35	NA
*CHL1-AS2*	−3.41	NA
*LINC02517*	−3.55	NA
*ENSG00000237596*	−3.72	NA
*ENSG00000289623*	−6.12	NA
*ENSG00000287592*	−3.61	NA
*ENSG00000254002*	−3.82	NA
*LINC01405*	−6.77	HNSC
*LINC01629*	−6.76	NA

**Table 4:** Hub genes detected in the co-expression analysis (degree > 30). Marine blue and orange text color indicates ion channel activity and long non-coding RNA, respectively.

Unique Name	Gene type	Degree
***NRXN3***	protein_coding	50
***TPH1***	protein_coding	47
***RTN1***	protein_coding	47
***PLD5***	protein_coding	45
***PCDH10***	protein_coding	45
***TRIM36***	protein_coding	44
***C8orf34***	protein_coding	44
***ACAN***	protein_coding	43
***ADAMTSL1***	protein_coding	42
***NT5DC3***	protein_coding	41
***CNTN1***	protein_coding	40
***MRAP2***	protein_coding	39
***GPR176***	protein_coding	39
***MYH10***	protein_coding	37
***ARPP21***	protein_coding	36
***CNTN4***	protein_coding	35
***GRIA2***	protein_coding	35
***HPSE2***	protein_coding	35
***RIMS1***	protein_coding	34
***CDH8***	protein_coding	34
***SUSD5***	protein_coding	33
***GABRR1***	protein_coding	33
***TMEM30B***	protein_coding	33
***PCDH11Y***	protein_coding	33
***C1orf87***	protein_coding	32
***CYTL1***	protein_coding	32
***CARTPT***	protein_coding	32
***IL31RA***	protein_coding	32
***PCDH11X***	protein_coding	32
***SAMMSON***	lncRNA	32
***ST6GAL2***	protein_coding	31
***DSC3***	protein_coding	31
***FOXCUT***	lncRNA	31

## Data Availability

The analytic results in this study are available on a request basis. All RNA-seq data we analyzed in the current study are uploaded into the Gene Expression Omnibus (GEO) database. GSE203367 and GSE226283 are accession numbers for RNA-seq on seven cardiac regions from sinus rhythm control and atrial fibrillation patients, respectively. GSE226282 is the accession number for RNA-seq on cut-off right atrial appendages.

## Supplementary Materials

The supporting information can be downloaded at: https://www.mdpi.com/article/10.3390/ijms241310501/s1.
